# SELMAP - SELEX affinity landscape MAPping of transcription factor binding sites using integrated microfluidics

**DOI:** 10.1038/srep33351

**Published:** 2016-09-15

**Authors:** Dana Chen, Yaron Orenstein, Rada Golodnitsky, Michal Pellach, Dorit Avrahami, Chaim Wachtel, Avital Ovadia-Shochat, Hila Shir-Shapira, Adi Kedmi, Tamar Juven-Gershon, Ron Shamir, Doron Gerber

**Affiliations:** 1 Mina and Everard Goodman Faculty of Life Sciences, Bar Ilan University, Ramat-Gan, 5290002, Israel; 2 Blavatnik School of Computer Science, Tel-Aviv University, Tel-Aviv, 69978, Israel

## Abstract

Transcription factors (TFs) alter gene expression in response to changes in the environment through sequence-specific interactions with the DNA. These interactions are best portrayed as a landscape of TF binding affinities. Current methods to study sequence-specific binding preferences suffer from limited dynamic range, sequence bias, lack of specificity and limited throughput. We have developed a microfluidic-based device for SELEX Affinity Landscape MAPping (SELMAP) of TF binding, which allows high-throughput measurement of 16 proteins in parallel. We used it to measure the relative affinities of Pho4, AtERF2 and Btd full-length proteins to millions of different DNA binding sites, and detected both high and low-affinity interactions in equilibrium conditions, generating a comprehensive landscape of the relative TF affinities to all possible DNA 6-mers, and even DNA10-mers with increased sequencing depth. Low quantities of both the TFs and DNA oligomers were sufficient for obtaining high-quality results, significantly reducing experimental costs. SELMAP allows in-depth screening of hundreds of TFs, and provides a means for better understanding of the regulatory processes that govern gene expression.

Transcription factors (TFs) are important components of gene regulatory networks. They alter gene expression in response to changes in the cellular environment^1^. Gene expression is controlled by TFs and co-factors, through their sequence-specific interactions with DNA. The analysis of transcription factor binding to DNA is best portrayed as a landscape of both high- and low-affinity binding sites^2^. Recently, technological advances have greatly increased our knowledge of the locations of TF binding sites within genomes and sequence-specific binding preferences for many TFs. These advances include both *in vivo* and *in vitro* experimental methods and the development of new methods of computational analysis^3^,^4^,^5^,^6^.

The most commonly used *in vivo* method for measuring TF-DNA interaction is chromatin immunoprecipitation (ChIP) (ChIP-chip and ChIP-seq). These methods are used to study the interactions between specific proteins and genomic DNA sequences by identifying occupied genomic regions^7^. In a ChIP experiment, the DNA-binding protein is crosslinked to DNA by treating cells with formaldehyde and shredding the chromatin by sonication into small fragments, generally in the 200–600 bp range. An antibody specific to the protein of interest is then used to immunoprecipitate (IP) the DNA-protein complex. Finally, the crosslinks are reversed and the released DNA is assayed to determine its sequences^8^. In ChIP-chip the chromatin IP is combined with a DNA microarray, while in ChIP-seq the resulting DNA fragments are sequenced^3^.

Despite the tremendous value of ChIP methods, they have technical limitations. The analysis requires the genomic DNA to be sheared into sized fragments that enable sequencing or loading into a microarray chip. In addition, a substantial amount of unbound DNA is trapped in the precipitate and generates a nonspecific signal. In many of these experiments, a bias in selection toward GC-rich fragments is observed, both in library preparation and in amplification prior to sequencing. Moreover, the potential of TFs to cross-react with other DNA-binding proteins present in the system may lead to imprecision in specific sequence determination^7^,^8^,^9^.

Several high-throughput *in vitro* techniques enable the measurement of relative binding affinities of a specific TF to many DNA sequences. These techniques greatly enhanced the extensiveness of characterisation of many known TFs. Protein binding microarrays (PBMs) use arrays of over 44,000 spots that together cover all possible 10-mer DNA sequences. Affinity measurements of 10-mers, each of which are present only once in the array, are insufficient for deriving conclusive results, so the 8-mer sequences, each occurring approximately 32 times on the array (taking both orientations into account) are used for the analysis. One advantage of PBMs is the ability to obtain semi-quantitative results, since the signal intensity within each spot on the microarray corresponds to the fraction of bound DNA-protein interaction. They can provide information about each DNA sequence variant and its relative binding preference. Nevertheless, PBMs have marked drawbacks: The assay is limited by the number of sequences that can be represented in a microarray, therefore, lower density microarrays have limited coverage of sequence space. In addition, the process requires several washing steps, which prevent detection of low-affinity interactions and measurements of protein-DNA interactions in equilibrium. Furthermore, binding measurements are limited to 10-mers, while it is known that for many TFs longer sequences are involved in DNA binding. Finally, the costly testing of human proteins on the microarray is a significant obstacle^10^,^11^,^12^,^13^,^14^,^15^.

Bind-n-seq is a single-step method in which one or more proteins are exposed to a library of DNA sequences, unbound oligomers are washed away while bound oligomers are sequenced and analysed for high-affinity motifs^16^. De novo binding preferences measured by this technology agree well with previous *in vitro* methods. Several potential binding sites can be recovered in each experiment. However, a single step may not always suffice for accurate detection of an affinity landscape of binding motifs.

Systematic evolution of ligands by exponential enrichment (SELEX)^17^ is an *in vitro* method that allows screening for specific ligand binding from a pool of all possible DNA sequences of a specific length^18^. SELEX methods have been used in the past to measure protein-DNA binding^19^,^20^,^21^,^22^, more recently in combination with high-throughput sequencing^12^,^23^. A critical step of SELEX is the removal of unbound DNA from the DNA–protein complexes. This often involves several washing steps that result in unintentional removal of weakly bound DNA, which cannot be controlled using conventional techniques. SELEX includes a gel retardation assay, affinity chromatography, a filter-binding assay, and other steps that complicate and prolong the process^10^,^24^. The success rate of testing full-length proteins is much lower than of DNA-binding domains (e.g. less than 12% compared to more than 25%, respectively, as reported in a recent study)^25^. Furthermore, several types of sequence bias were reported for this technology^14^.

Despite the development of high-throughput methods, our understanding of the interconnections between transcriptional regulators and their targets is still incomplete. Current methodologies for characterising DNA-protein interactions suffer from limited dynamic range, allowing for detection of only the most strongly bound motifs. As a result, weaker regulatory interactions other than those occurring at high-affinity binding sites are largely ignored and are not well understood^26^.

Recently, a novel method for studying DNA-protein interactions has been developed, based on programmed microfluidic devices^27^,^28^,^29^. The assay introduces several advantages compared to the currently used methods. The microfluidic assay eliminates the need for high levels of protein expression and purification, allowing for low costs of the experimental procedure. Furthermore, application of the microfluidic platform enables the use of smaller reaction volumes, reducing the amount of DNA used in each experiment and increasing the DNA concentration accessible for the TFs to induce interaction. A “snapshot” of the equilibrium created is achieved using mechanically induced trapping of molecular interactions (MITOMI), enabling the detection of weak protein-DNA interactions. This provides means for determining binding specificities through direct measurements of binding affinities to thousands of different DNA sequences per device^27^,^30^. In addition, the microfluidic device offers the advantage of screening many TFs in parallel, and can therefore be used in a high-throughput fashion with respect to both the DNA and the proteins^31^.

In the current work, previously studied TFs were immobilised onto the surface of a microfluidic device, and their consensus sequences as well as low-affinity binding sequences were bound and isolated from a large library of sequences by a SELEX procedure. The first TF was a well-studied *Saccharomyces cerevisiae* TF, regulatory protein phosphate system positive regulatory protein (Pho4)^32^,^33^. The second was *Arabidopsis* thaliana AtERF2 protein, a member of the ethylene-responsive element binding factors (ERFs) family^34^,^35^. Quantitative analysis of bound DNA sequences was achieved by high-throughput sequencing (HTS). The binding affinity landscaping of both strongly- and weakly-bound oligomers for different TFs on a microfluidic chip was successfully demonstrated. We also report the first high-throughput measurements of the DNA-binding preferences of *Drosophila* Btd, an Sp family member Zinc finger TF, in its full-length version. SELEX Affinity Landscape MAPping on a microfluidic platform (SELMAP) allowed for 16 parallel assays, increasing dynamic range and lowering experimental costs, compared to existing methodologies. This highlights the potential of microfluidics in high-throughput screening for a landscape of binding affinities of large numbers of TFs simultaneously.

## Results

### Design of the 12-mer library

**Figure 1:**
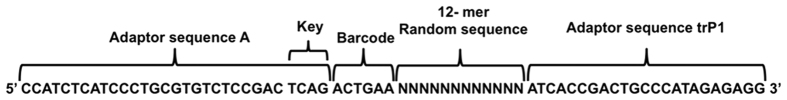
Oligomer template design. The template includes adapter sequences A and trP1 for incorporation into the HTS instrument. Adaptor A includes a “key” for instructing the instrument to begin the read. The adaptors were also used for hybridisation to PCR primers during amplification. The barcode was used for the library identification and was unique for each library. The 12-mer random sequence potentially includes all possible 4^12^ sequences to be screened for TF binding.

SELEX experiments were performed using a large library of DNA oligomers to measure the relative TF-DNA binding affinities. Each double stranded DNA oligomer was composed of five segments: an adapter sequence A, a ‘key’ segment, a ‘barcode’, a focal 12-mer random sequence, and a second adapter sequence trP1, resulting in a 71 bp long sequence (Fig. 1). The pair of ‘adaptors’ were used for hybridisation to the solid support for the HTS reaction^36^, and were also employed as the hybridising segment to the real-time quantitative PCR (qPCR) primers. The barcode was used for identification of the origin of the sequence from parallel experiments within the microfluidic chip. Two 12-mer libraries were obtained and labelled with a unique barcode for identification purposes, which in turn was designated to a specific TF. The ‘key’ segment allowed for the alignment of all reads and identification of where each insert begins by the HTS software.

To test the uniformity of the initial library in terms of nucleotide composition, we calculated the Kullback-Liebler divergence (KLD) score for 6-mers (see Methods). KLD_6_ was 0.05 for library #1, and 0.037 for library #2. A perfectly uniform library would have KLD = 0, and the maximal possible KLD value is 2. Initial oligo libraries with KL-divergence of up to 0.12 were used successfully in HT-SELEX^37^. Hence, both libraries were of high quality and had a near-uniform 6-mer distribution.

### One protein-one library study

**Figure 2:**
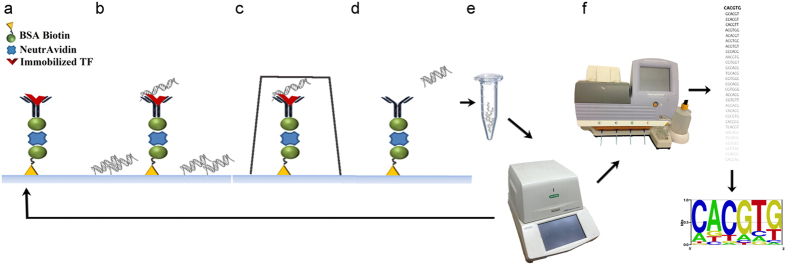
SELMAP assay for a single protein within a microfluidic chip. An illustration of the SELMAP experimental protocol. (**a**) The TF is bound to its antibody beneath the button in the microfluidic chip. (**b**) The oligomers comprising the 12-mer library are then flowed through the chip and both specific and non-specific binding occurs. (**c**) The button is applied, high- and low-affinity oligomers remain bound to the TF and non-specifically bound DNA is degraded and washed away. (**d**) The TF is then degraded with a protease, releasing the bound oligomers. (**e**) The released DNA is eluted, collected and amplified and (**f**) a sample of the DNA is sequenced by HTS, and then analysed to infer affinity scores for all DNA 6-mers. This procedure is repeated for each enrichment round (**a**–**e**).

As an initial proof of concept, a 12-mer library was exposed to a single TF. The 12-mer library was loaded into a microfluidic chip with pre-bound TF, Pho4. By closing the button valves, we trapped different 12-mer sequences with varying affinities to Pho4, generating a “snapshot” of the interactions at equilibrium. Non-specifically bound oligos (not under the button) were degraded with endonuclease. The TF was subsequently degraded with protease in order to release the bound oligos into solution, which were then eluted from the entire chip and amplified by real-time PCR. This procedure was performed with 3 enrichment cycles and the data from each round was sequenced by HTS and analysed by appropriate software (Fig. 2).

**Figure 3:**
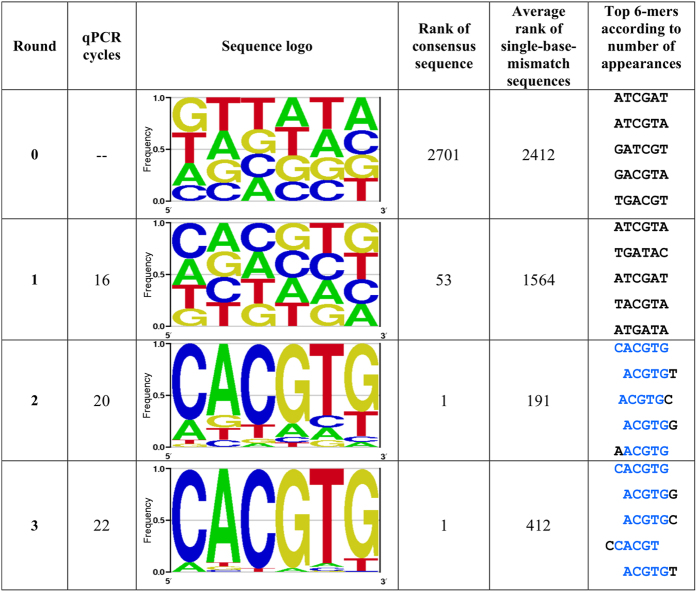
Summary of enrichment rounds of one protein one library experiment. The optimal number of qPCR cycles was determined to be the minimal number of cycles in the exponential phase of amplification above the threshold of fluorescence detection (see Methods). The amplified sample was used as the DNA input for the next round of enrichment. Each round was sequenced and analysed. The sequence logo represents single-mismatch variations for the given consensus (CACGTG) based on observed 6-mer frequencies. Optimal enrichment in this case is observed after two rounds. In the third round we observe that the consensus sequence overrides the available sequence space, narrowing the dynamic range. The higher the ranking of the 6-mers, the stronger the relative binding of Pho4 to the sequences.

Each enrichment round resulted in increased specificity of the TF towards the 12-mer library, leading to a narrowed library and changes in relative concentrations of eluted 12-mer sequences. The eluted sequences were used to compute the observed frequency of each 6-mer (within the 12-mer library) indicating its relative binding strength to the TF. Three different binding scores were calculated for each DNA 6-mer in each cycle: frequency (i); the ratio of its frequency to that of the previous cycle (i/i-1); and the ratio of its frequency in round i to that in the initial cycle (i/i-0). The set of all 6-mers together with their binding scores constitute a comprehensive model of the protein binding preferences. The position weight matrix (PWM) derived from the sequencing data analysis is produced for visual interpretation (see Methods). PWMs were derived from the seed sequence and 6-mers at one Hamming distance from it (see Methods). Sequencing results of the initial library (“round 0”) and each round of enrichment are summarised in Fig. 3.

The number of qPCR amplification cycles required for an optimal signal was determined for each round (see experimental section and Supplementary Fig. S1). The experiment involved the use of DNase and washing steps that eliminate DNA that is not bound under the button, decreasing non-specific binding and resulting in elution only of the 12-mers that interact with the TF. The concentrations of DNA eluted appeared to vary slightly from cycle to cycle. As a control experiment, we performed SELEX comparing DNA binding to Pho4 to DNA binding within the device without a TF (on a single chip divided into two). DNA bound to Pho4 was eluted in significant quantities, as observed by qPCR (Ct generally below 22 PCR cycles), whereas the negative control observed in much lower quantities (Ct generally above 22 PCR cycles, compared to HPLC grade water, with Ct~30 cycles). The number of PCR cycles performed for enrichment of the specifically bound product was kept below 22 cycles, prior to any enrichment of non-specifically bound DNA (See Supplementary Fig. S2).

Several sequence biases have been previously reported for HT-SELEX^14^. In order to test for their presence in SELMAP, we counted the number of oligos that do not contain CACGTG among the 100 most frequent oligos in round 3. Only two were detected, so false oligo bias seems very minor or nonexistent. In addition, no enrichment of C-rich k-mers was observed through the cycles. CACGTG had a ratio score of 220.25 in the last round, compared to 1.66 for CCCCCC, where the mean ± std was 1.11 ± 4.63. Further testing on larger datasets is needed to check for other biases.

The enrichment procedure described above successfully identified the specific DNA sequences that bind to Pho4 transcription factor (see Fig. 3). In the first round, the algorithm could not detect correct binding to Pho4 due to relatively low enrichment of the consensus sequences, which are still shadowed by the initial library frequencies. Nonetheless, a closer look at the data reveals that the consensus sequence ‘CACGTG’ was initially positioned 2701^st^, and following round 1 moved to the 53^rd^ position, indicating enrichment of specifically bound sequences. In rounds 2 and 3, the 6-mer consensus sequence, CACGTG, was already the most frequently occurring sequence and dominated the sequence population, having highest affinity to Pho4. The single-base-mismatch 6-mers were also strongly bound by Pho4. However, the landscape spectrum of DNA binding affinities at the 3^rd^ round was of lower quality, since the consensus sequence overpowered the single-base mismatches. In the case of Pho4 two rounds of enrichment were found to be optimal, and without loss of affinity landscape information.

**Figure 4:**
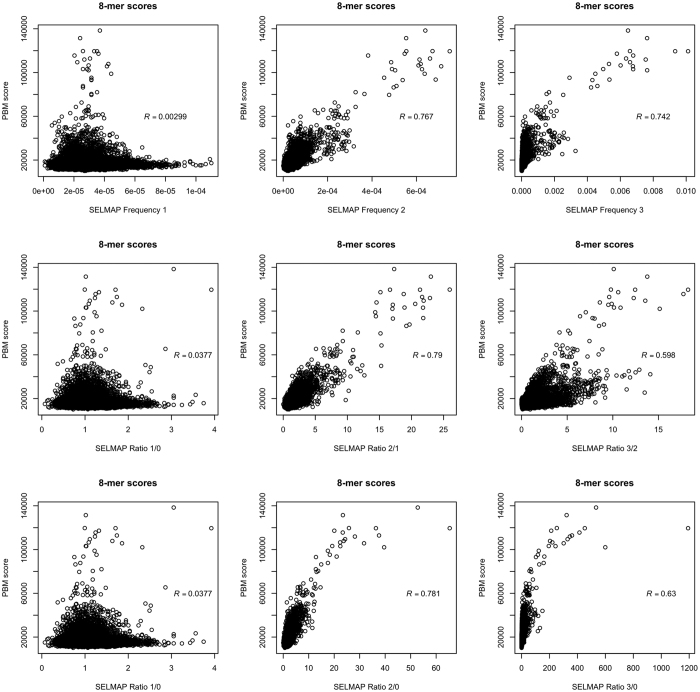
Pearson correlations between PBM and SELMAP data. High correlations between SELMAP data from this study and published PBM data from UniPROBE were observed for both rounds 2 and 3 of enrichment. Pearson correlation was calculated based on all 32896 unique 8-mers scores. PBM scores were based on average binding intensities. Different subplots correspond to different SELEX scores (x-axis).

In order to validate our method, we compared our results with published PBM data^38^. We derived scores for all possible 8-mers by averaging the score of all sequences in which they appear (see Methods). We calculated Pearson correlations between the 8-mer scores derived from our experiment to those derived from PBM data. A strong correlation to PBM experimental data was achieved after the second enrichment round. (Fig. 4).

### Reducing the sample size to allow parallel experiments

A smaller sequence sample size would allow for smaller space to be utilised on the chip per experiment, allowing for multiple experiments to be performed on a single chip. The concern was whether it was possible to obtain sufficient concentrations of DNA, when the samples were taken from a smaller chip area. Enrichment rounds were performed with the initial DNA 12-mer library #1 and with Pho4 as the binding protein. DNA samples collected separately from 1, 2, 4 and 8 columns (out of the 16 columns of the chip), and the samples were amplified by qPCR. The standard curve from round 1 of each collection was examined and showed no significant differences between samples. The collected sample from a single column of chip required a similar number of amplification cycles compared to samples from 2, 4 and 8 columns. Therefore, elution of sufficient quantities of DNA was achieved from the single chip column, and potentially, the number of samples that can be analysed in parallel depends on the number of columns in a device, which in our case was 16. With the development of different chips comprising hundreds of different proteins, each transcription factor could be screened simultaneously with individually barcoded oligo libraries, and the procedure could become beneficial for high-throughput screening.

### Simultaneous SELMAP binding affinity measurements of multiple TFs

**Figure 5:**
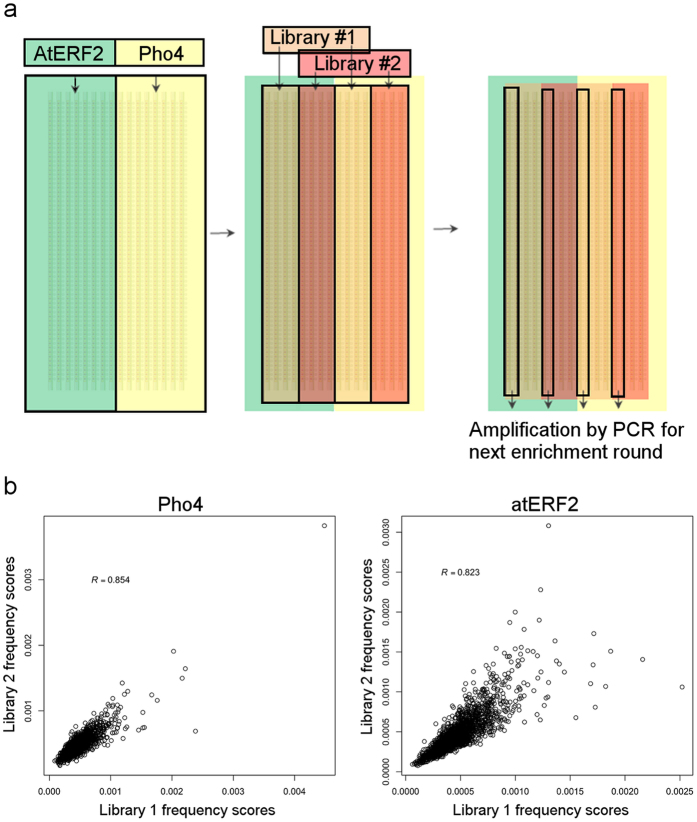
Experimental design and reproducibility. (**a**) Allocation of columns of the microfluidic chip to simulate 16 parallel measurements. The chip was divided in half for each of the TFs Pho4 and AtERF2. The 12-mer random libraries flowed through the microfluidic chip such that each library was directed to both halves of chip. Non-specifically bound DNA was degraded by endonuclease. Specifically bound DNA was released by TF degradation with proteinase K. DNA from just a single column of each quarter (a single measurement from each) was collected and amplified. The procedure was repeated before HTS. (**b**) 6-mer scores of round 2 frequencies. The frequency scores of two parallel experiments on the same protein demonstrate the high reproducibility of our experimental design.

The feasibility of measuring several proteins simultaneously was demonstrated with two proteins and two oligo libraries. One half of the chip was loaded with Pho4 while the other half was loaded with AtERF2. The two 12-mer libraries were then flowed in such a way that each protein was able to interact with each library separately, giving 4 possible combinations: Pho4 interaction with library #1, Pho4 with library #2, AtERF2 with library #1 and AtERF2 with library #2. This arrangement simulated sixteen parallel measurements. Each quarter of chip (four of sixteen columns) was allocated to each protein-DNA combination. DNA was eluted from just a single column of each combination, amplified and reintroduced to the next chip containing the two expressed TFs in the same manner, as illustrated in Fig. 5a. Again, following the second enrichment round, DNA from a total of only four columns was collected. Based on the results of the “one protein one library” experiment, in which the optimal results were obtained after two enrichment rounds, DNA oligonucleotides eluted from the second round were sequenced and analysed. As mentioned, each library was marked with different barcodes, enabling determination of the origin of the reads.

**Figure 6:**
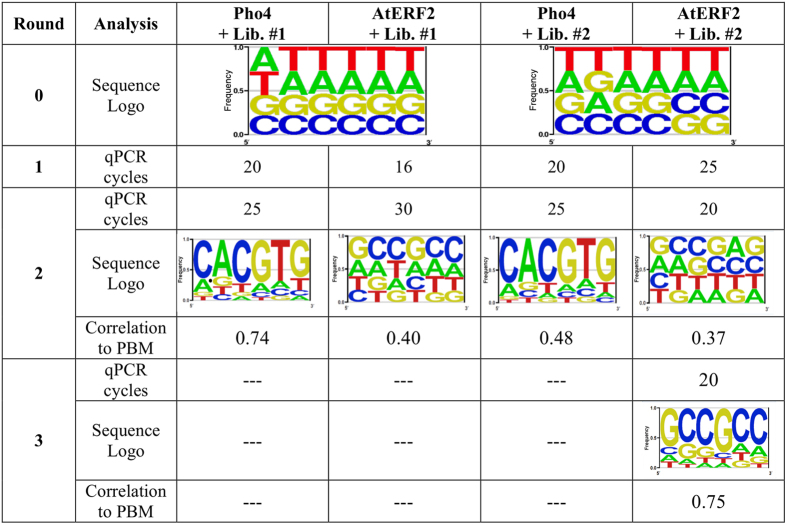
Summary of enrichment rounds of high-throughput TF-binding. Round 0 of enrichment of library #1 and library #2 are the initial DNA libraries applied to the chip. In round 1, optimal amplification cycles were determined and applied in each round. The amplified sample was used as the DNA input for the next round. The sequence logo represents the derived consensus and single-base-mismatch motifs. For round 0 the PWM is based on all 6-mers. A third enrichment round was performed only for the AtERF2 experiment with library #2, which after the 2^nd^ enrichment round did not display the consensus sequence as the highest affinity sequence.

The relative amount of oligos that were derived from a single column was smaller than that of the first experiment conducted on Pho4 only (16 columns), which explains the higher number of required amplification cycles (see Fig. 6). In each round, before sequencing, the eluted 12-mers from the two pho4 and two AtERF2 columns of the chip were combined and sequenced simultaneously. The barcodes allowed for unique identification of the libraries, as explained earlier. To gauge reproducibility, we calculated the Pearson correlation between 6-mer frequencies in parallel experiments (see Fig. 5b).

A landscape of binding affinities for each of the four protein-DNA combinations was successfully elucidated. The sequence logos represent the highest-affinity 6-mers of each TF-DNA 12-mer library combination after round 2 or 3 of enrichment. High correlations in binding affinity landscapes were observed between Pho4 interactions with library #1 (Fig. 6), and the previous ‘one protein one library’ experiment (Fig. 3). Exposing AtERF2 to library #1, the 6-mers with highest affinity contained the consensus sequence GCCGCC^35^,^39^, as well as related sequences that were ranked closely after. Following the second enrichment round of interaction between AtERF2 and library #2, the consensus sequence appeared at the 38th position compared to 4041st prior to enrichment (“round 0”). Following a third enrichment round, the consensus sequence was the highest ranked sequence in terms of affinity, with closely related sequences showing weaker affinity but still with specificity towards the TF, in a similar manner to our Pho4 results. These results demonstrate that a landscape of TF-binding affinities can be captured by two or three enrichment cycles.

### Accuracy of detection of low-affinity binding using SELMAP and PBM

To compare the ability of detecting low-affinity binding by SELMAP on a chip with PBM, we used published measurements of Pho4 binding probabilities to synthetic promoter sequences^40^. The promoter sequences included two binding sites, one exposed to Pho4 binding and the other occluded by a nucleosome. Binding probabilities were computed from binding energy measurements. We used promoter sequences with mutations in the core consensus 6-mer site only, where only one of the two sites was mutated. This allowed an unbiased comparison to 6-mer scores generated by SELMAP and PBM. For SELMAP, we preferred the frequency scores from cycle 2 over those from cycle 1, as 6-mer scores from cycle 2 showed a highly-enriched consensus sequence while not overshadowing non-consensus bases. For PBMs we used the average binding strength. To measure the accuracy, we calculated the Pearson correlation between published binding probabilities of 6-mers^40^ and their PBM and SELMAP scores.

**Figure 7:**
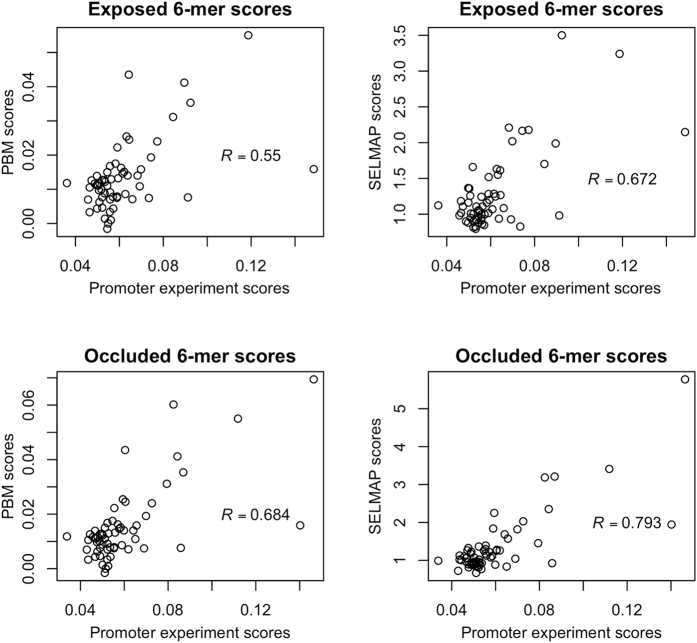
Correlation of PBM and SELMAP binding scores to experimentally validated promoter binding sites. For exposed and occluded binding sites, accurate binding intensities were calculated previously. We compared these intensities to PBM- and SELMAP-based binding scores. For PBM we used average binding intensities, and for SELMAP ratio of frequency in round 2 over round 1.

Using 60 6-mers available at the exposed binding site, the correlation was 0.67 for SELMAP compared to 0.55 for the PBM (p-value = 0.05). For 62 available 6-mers in the occluded region, the correlation was 0.79 for SELMAP compared to 0.68 for PBM (p-value = 0.007) (See Fig. 7). We note that for other 6-mer scores, (e.g. per-round frequencies or frequency ratios at other rounds) SELMAP did not show improved correlation. These results indicate that on this dataset SELMAP gives more accurate measurements of binding affinities to low-affinity sites compared to PBM.

### Longer Motif detection

**Figure 8:**
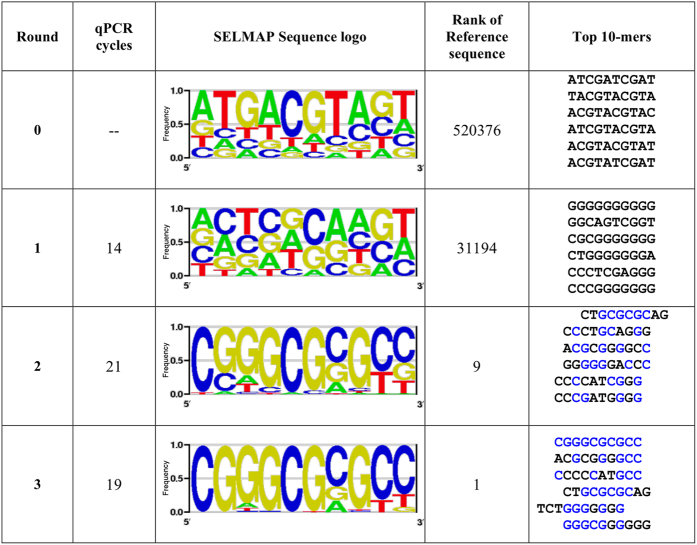
Summary of three rounds of enrichment of sequences that bind to Btd. A sequence logo was generated for each round of the SELMAP assay performed using Btd. Two enrichment rounds allowed for generation of a CG-rich 10-mer sequence logo, further enriched in round 3, demonstrating its high affinity for Btd. The top 10-mers are listed according to the ratio of their frequency to the estimated ratio in the initial cycle. The reference sequence in column 4 is CGGGCGCGCC.

To demonstrate binding measurements for longer sequences, we used the *Drosophila melanogaster* Buttonhead (Btd) transcription factor. Btd is an Sp family Zinc finger transcription factor that binds a GC-rich DNA sequence^41^,^42^. This previously known binding preference was based on 32 binding sites detected by bacterial one-hybrid (B1H) platform^43^ and a recent HT-SELEX experiment^25^. In both previous B1H and HT-SELEX experiments, only the DNA-binding domain was tested. In order to discover the full-length version of the Btd affinity landscape to all possible DNA 10-mers, we performed three SELMAP rounds with deeper sequencing coverage compared to the previous experiments (more than 2 M reads per round compared to less than 500 K). From these data we derived all possible 10-mer binding scores, where we estimated the initial cycle frequencies using a 5^th^-order Markov model due to insufficient read coverage, as done in the SELEX-seq protocol^24^. Throughout the SELMAP procedure, results produced a clear enrichment of 10 bp long GC-rich sequences (Fig. 8).

**Figure 9:**
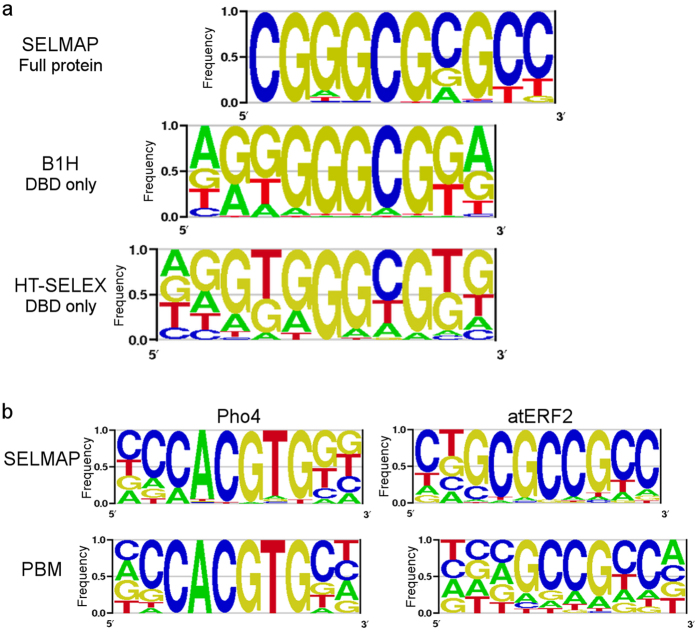
10-mer sequence logos obtained using SELMAP compared to other methodologies. (**a**) Btd-binding sequences obtained using SELMAP, B1H and HT-SELEX. Common to all sequence logos is the GGGCG motif, found in positions 4–8 using B1H, positions 5–9 using HT-SELEX, and shifted with SELMAP to positions 2–6. The differences in the flanks are likely due to the fact the full-protein was tested in SELMAP compared to only the DNA-binding domain (DBD) tested by B1H and HT-SELEX. (**b**) 10 bp-long Pho4- and AtERF2- binding sequences, derived using SELMAP and PBM. For Pho4, using the SELMAP method, the reported consensus CCCACGTGGG was detected, whereas previously reported PBM results lacked some of the core flanks. For atERF2 the PBM’s flanks have uniform frequencies, while SELMAP gives more informative ones. Hence, while SELMAP can accurately identify binding preference for positions flanking the core, PBM is limited to accurately measuring 8 positions. PBM-derived PWMs for Pho4 and AtERF2 were downloaded from CIS-BP (motif IDs M0242_1.02 and M0038_1.02, respectively). SELMAP motifs were based on round 3 data for Btd and AtERF2, and on round 2 for Pho4.

By comparing our results with those obtained from the B1H and HT-SELEX experiments (Fig. 9a), we observe that the core of the sequence (GGGCG) is consistent using all methods. However, nucleotides 7–8 in the logo produced using SELMAP differed from those in the corresponding positions (9–10 and 10–11) of the logos found using B1H and HT-SELEX, respectively, and nucleotides 9–10 in the SELMAP results had no match for comparison in those of B1H and HT-SELEX. This discrepancy was observed in round 2 and was further enriched in round 3, which confirmed their affinity to the TF. We note that sequence logos are dependent on the methods used to derive them and it is preferable to compare k-mer scores, but at this point the read coverage of HT-SELEX experiments does not allow the inference of accurate k-mer scores^14^ (slightly more than 200 K in the last round compared to more than 2 M in SELMAP). They differ mostly in the flanks rather than at the core. We believe that the differences in binding preferences measured by SELMAP compared to B1H and HT-SELEX mostly result from the fact that the full-protein version was tested compared to only the DNA-binding domain.

To show that our analysis of longer motifs can be applied more generally, we derived 10-mer binding scores for Pho4 and AtERF2 from experiments that had sufficient sequencing depth. Our original Pho4 experiments included more than 500 K sequence reads in round 3 and AtERF2 2-library experiments included more than 2 M reads in round 3. For Pho4, CCCACGTGGG appeared as the highest-ranking 10-mer, in concordance with a previous study that measured the effect of flanks on Pho4 binding^30^. For AtERF2 we discovered previously unidentified binding preferences to the flanks of the core GCCGCC, and a highest-ranking 10-mer: CTGCGCCGCC. Future studies using other techniques are needed to validate AtERF2 binding preferences to the flanks. The data from PBM, on the other hand, were insufficient for resolving the flanking preferences (Fig. 9c).

## Discussion

Using microfluidics, we developed a new experimental method to measure the binding preferences of multiple proteins to thousands of DNA oligos simultaneously. Moreover, we demonstrated that the consensus sequences that are specifically bound by each of two well-studied TFs, Pho4 and AtERF2, could be isolated from a large random library of oligomers, amplified and detected by SELEX procedures. The unique microfluidic setup allowed not only for isolation and detection of the consensus sequences, but also for deriving a landscape of binding affinities including both high and low affinity DNA 6-mers, and even 10-mers at greater sequencing depth. This was achieved due to an equilibrium that was created by a highly-controlled flow of a constant concentration of the 12-mer DNA library, not possible with other SELEX-like procedures. The presence of the “buttons” allowed for a “snapshot” of the equilibrium between the TF and relatively weakly-bound sequences. This MITOMI technology also allowed for degradation of DNA by endonucleases and thorough washing of non-specifically bound DNA. In addition, protein expression *in situ* allowed for successful measurement of Btd full-length protein and discovery of its binding preferences, which differ from the DNA-binding domain.

In the binding studies of Pho4, with both libraries and using both the larger and smaller chip volumes, two enrichment rounds were required in order to give an overriding sequence, confirmed to be the consensus sequence. In the case of AtERF2, however, a third enrichment round was required. It is known that the Kds of binding sequences for AtERF2 with the GCC box motif range from the picomolar to the micromolar levels^34^. Perhaps due to this high variability, there is a need for the additional enrichment round for the consensus sequence to overcome the presence of other sequences.

Assessment of the quality of the affinity scores obtained was based on the Pearson correlation between PBM 8-mer scores to our SELMAP 8-mer scores. A correlation of 0.74 was achieved for the third AtERF2 enrichment round despite the fact that a lower depth of sequencing was performed. This correlation was considered to be quite high, taking into account the fact that the scores came from independent experimental platforms using different technologies, showing that high-quality results could be obtained despite a relatively low depth of sequencing. Relatively low correlation was observed for the interaction of Pho4 with Library #2. Although the correlation of the former is significantly lower compared to the latter, overall, the landscape of binding affinities appears to be quite similar for Pho4 for both libraries. In addition, while PBM data is a valuable guide for data validation, there is also a possibility that in many cases the accuracy of screening by SELEX on a microfluidic chip exceeds that of the PBMs, with larger sequence space and inclusion of low-affinity binding. Indeed, in our case the SELMAP had higher accuracy than PBM for evaluating low-affinity TF-6-mer binding.

In this study, we derived, for the first time in high-throughput, the affinity landscape of Btd in its full-length to all DNA 10-mers. Results correlated well with known general preferences of Zinc finger proteins to G/C-rich sequences, as well as the core binding motif derived by B1H and HT-SELEX protocol. SELMAP found binding preferences for the flanks of the core that were different from both B1H and HT-SELEX, which showed high similarity in their motif logos. Since SELMAP tested the full-length protein, compared to B1H and HT-SELEX, which test the DNA-binding domain, we believe that SELMAP was able to recover binding preferences that are more relevant biologically as *in vivo* the protein is expressed in its full-length form. These newly discovered preferences could benefit the gene regulation research community. The conclusion that full-length proteins may have different binding preferences from their DNA-binding domains alone indicates a need to experimentally-measure binding preferences of full proteins. Moreover, we recovered known binding preferences of Pho4 to motif flanks, and discovered novel preferences of AtERF2.

Overall, the parallel study of two TFs with two large oligomer libraries was used to demonstrate the possibility of simultaneous measurement of sixteen TF binding preferences. The SELEX technique offered the possibility of full screening of all possible 12-mer DNA oligos, and it was demonstrated that results could be obtained after just two or three enrichment rounds. The experiments were performed with low concentrations of DNA, and with low volumes of solution, allowing for additional simultaneous experiments using the same microfluidic chip for each round, and at lower costs compared to existing SELEX-like technologies. Notably, we have successfully measured the DNA binding preferences of TFs from three different organisms containing three different types of DNA binding domains (bHLH, AP2 and zinc finger domains). Thus, the system provides a means for analyzing TFs from multiple/diverse organisms. With future development of methods for preparing a chip with large numbers of columns, each TF could be screened simultaneously with individually barcoded oligo libraries. We have thus demonstrated the potential for future high-throughput parallel screening of a large number of proteins, and characterisation of their landscape of DNA binding affinities.

## Methods

### Chip fabrication

The microfluidic device was fabricated in a manner similar to that previously described^44^,^45^. Briefly, fabrication was performed on silicone molds casting silicone elastomer polydimethylsiloxane (PDMS, SYLGARD 184, Dow Corning, USA). Each device consists of two aligned PDMS layers, the flow and the control layer. The molds were first exposed to chlorotrimethylsilane (Aldrich) vapour for 10 min to promote elastomer release after the baking steps. A mixture of silicone based elastomer and curing agent was prepared in two different ratios 5:1 and 20:1 for the control and flow layers, respectively. The control layer was degassed and baked for 30 min at 80 °C. The flow layer was initially spin coated (Laurell, USA) at 2000 rpm for 60 sec and baked at 80 °C for 30 min. Next, the flow and control layers were aligned manually under a stereoscope and baked for 1.5 h at 80 °C (See Supplementary Fig. S3), for final adhesion.

### Immobilisation of TFs

Surface chemistry was implemented, inside the chip, on the epoxy layered slide by flowing Biotinylated-BSA (1 μg/μl, Thermo) for 20 minutes, followed by Stepavidin (Neutravidin, Pierce, 0.5 μg/μl) for 20 minutes. The ‘Button’ valves were closed and a second dose of Biotinylated-BSA (1 μg/μl, Thermo) was introduced for 20 minutes, passivating all areas surrounding the ‘Button’ valves. Following passivation, the ‘button’ valves were released and a flow of penta-His Biotinylated antibody (Qiagen, 0.2 μg/μl) allowed the antibody binding directly beneath the button. His-tagged Pho4 (UniProt accession no. P07270, with a basic helix-loop-helix (bHLH) binding domain at positions 250–306,) or AtERF2 (UniProt accession no. O80338, with an ERF (AP2 family) binding domain at positions 116–174) (12.5 μl) were expressed *in vitro* using rabbit reticulocyte quick coupled transcription and translation reaction (TNT, Promega) with 0.5 ul of fluorescently labelled lysine (FluoroTect™ GreenLys, Promega CAT #L5001). Btd (UniProt accession no. Q24266, with zinc finger DNA-binding domain at positions 333–357, 363–385 and 391–413) was cloned using cDNA prepared from 0–12 hrs *Drosophila melanogaster* embryos in frame of C-terminal V5 and His tags into the pAc5.1V5His expression vector (Life Technologies). Plasmid sequences were verified by sequencing and Btd was overexpressed in *D. melanogaster* Schneider S2R + adherent cells (see Supplementary Information and Fig. S4). The TFs were introduced into the microfluidic device and immobilized to the slide surface beneath the ‘button’ valves.

### SELMAP assay

Two ssDNA oligos were annealed slowly for 20 min after heating to 95C for 5 min. the resulting 71-bp dsDNA comprising a random 12-mer library (IDT, 20 μl, 50 μM) was flowed through TF-loaded chip for 20 min. The button valves were closed, and surrounding unbound DNA was degraded using DNase (20 μL, 200 units/mL New England Biolabs). The DNase was then inactivated by heating the chip to 75 °C using a hot plate for 10 minutes and washed away with phosphate buffered saline for 10 minutes. Afterwards, Proteinase K (100 μg/ml, Halt™ Protease Inhibitor Cocktail, Thermo Scientific) was added and incubated on the chip with open button valves for 30 minutes at 50 °C. The remaining oligonucleotides were collected for amplification by PCR (together with the degraded TFs) using double-distilled water. The optimal number of PCR cycles was determined according to the fluorescent signal intensity for each amplification cycle (using SYBR^®^ Green FastMix ROX, Quanta Biosciences), plotted on a standard curve. This was set for each experiment as the minimal number of cycles in the exponential phase of the PCR process, in order to reduce PCR-induced biases (see Fig. S1, S2)^46^. The collected DNA was amplified using the pre-determined optimal number of cycles, by qPCR (CFX96 BioRad). After PCR the DNA was either sequenced by HTS (Ion Torrent™, Life Technologies or Illumina MiSeq^**®**^) and analysed, or subjected to a subsequent enrichment round on an additional chip loaded with TF and subsequently sequenced.

### HTS Data analysis

Data analysis was implemented on DNA products recovered from high-throughput sequencing. The Ion semiconductor chip detects polymerase-driven base incorporation and translates this information into digital form. The number of reads per chip was 1–1.5 million. Sequencing results were encoded in a text-based FASTQ format, for storing both a nucleotide sequence and its corresponding quality scores^47^. Raw sequencing files are freely available on http://www.ebi.ac.uk/ena/data/view/PRJEB9897.

### Measuring uniformity of initial oligo library

To measure the uniformity of the initial oligo library, we used the KLD score^48^. The score measures the distance in bits between two distributions. In our case, one distribution is the observed k-mer frequencies and the other the uniform distribution, i.e., each k-mer has a 1/4^k^ probability of occurring in the sequence pool. The score has been successfully used on SELEX-seq data^23^. Formally, given *k* and vector *f*_*i*_ of the observed k-mer frequencies, the score is expressed as:


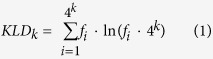


### Computational analysis

We implemented a software tool to analyse the data and generate k-mer scores. The software receives as input k, the barcode specifying the relevant sequences, expected oligo length, seed to generate a PWM by (see below) and sequencing files. The tool first filters out sequences without the barcode, containing an unidentified nucleotide “N” or of the wrong length. The number of occurrences of each k-mer in each cycle are counted in the remaining sequences. Using these counts, the tool generates 3 different affinity scores for each k-mer in each cycle, in a similar manner to as previously described^14^. 1. f_i_ (w) = the frequency of k-mer w in cycle i; 2. r_i_ (w) = f_i_ (w)/f_i−1_ (w) = ratio of the frequency of k-mer w in cycle i to its frequency in the previous cycle; and 3. r_i0_ (w) = f_i_ (w)/f_0_ (w) = the ratio of the frequency of k-mer w in cycle i to its frequency in the initial round. For 10-mer analysis, we replaced the frequencies in the initial round by estimated frequencies (using 5^th^-order Markov model, as in SELEX-seq^24^). The software and processed data are freely available on acgt.cs.tau.ac.il/selmap/.

### PWM generation

PWMs were generated for visual interpretability. A PWM was generated based on a given consensus seed. The top-ranking 6-mer/10-mer in the last cycles was chosen as seed (CACGTG, GCCGCC and CGGGCGCGCC for Pho4, AtERF2 and Btd, respectively). For a given seed, all k-mers at Hamming distance ≤1 from it in the sequence data were collected and aligned, the frequency of each nucleotide was computed in each column, and the values in each column were normalized to probabilities. This approach was originally used for HT-SELEX data^12^. PWMs were plotted using: http://lagavulin.ccbb.pitt.edu/cgi-bin/enologos/enologos.cgi 49.

### Validation with PBM data

To validate SELMAP experimental results we compared them to results of PBM experiments performed on the same proteins. The Pho4 and AtERF2 results were downloaded from UniPROBE^50^ and CIS-BP databases^39^, respectively. 8-mer scores were extracted from each dataset. For PBM, each 8-mer was assigned its average binding score, which was shown to provide a robust and accurate score for such data^13^. The similarity was measured using Pearson correlation coefficient between the vectors of 8-mer scores.

### Comparison of SELMAP and PBM in measurements of low-affinity binding

We used 6-mer scores from a study measuring Pho4 binding to synthetic promoter sequences^40^. Rajkumar *et al.* measured binding probabilities of Pho4 to synthetic promoters containing exposed and occluded (nucleosomal) sites. In each promoter, mutated versions of the consensus binding site were introduced in either the nucleosomal or exposed site. Of those, we analysed the sites that contained mutations in the consensus and none in the flanks, and in only one of the two sites, totalling 60 exposed and 62 nucleosomal binding sites. Measured differences in energy affinities ΔΔG were transformed to binding probabilities using the transformation 1/(1 + exp (ΔΔG*0.592), as described in the original study. Pearson correlation was calculated between these 6-mer probabilities and their SELMAP freq (2)/freq (1) scores, and between the 6-mer probabilities and their PBM average binding intensity scores, separately. P-values to compare correlation coefficients were calculated using http://quantpsy.org/corrtest/corrtest2.htm 51.

## Additional Information

**How to cite this article**: Chen, D. *et al.* SELMAP - SELEX affinity landscape MAPping of transcription factor binding sites using integrated microfluidics. *Sci. Rep.* **6**, 33351; doi: 10.1038/srep33351 (2016).

## Supplementary Material

Supplementary Information
