# Supplementary material for: SELMAP - SELEX affinity landscape MAPping of transcription factor binding sites using integrated microfluidics
Source: Sci Rep. 2016 Sep 15;6:33351. doi: 10.1038/srep33351 (PMC5024299; doi:10.1038/srep33351)
Supplement: Supplementary Information [file srep33351-s1.pdf]

SELMAP - SELEX affinity Landscape Mapping of transcription factor binding sites using integrated microfluidics

Dana Chen, Yaron Orenstein, Rada Golodnitsky, Michal Pellach, Dorit Avrahami, Chaim Wachtel, Avital Ovadia-Shochat, Hila Shir-Shapira, Adi Kedmi, Tamar Juven-Gershon, Ron Shamir, Doron Gerber*

Supplementary Information

1. Supplementary experimental section:

Transient transfections and preparation of cell extracts containing the Buttonhead (Btd) transcription factor. *D. melanogaster* Schneider S2R+ adherent cells were cultured in Schneider's *Drosophila* medium (Biological Industries) that was supplemented with 10% heat-inactivated FBS. For Btd overexpression, cells were plated at 3×10^6 cells per 60mm dish, one day prior to transfection. Cells were transfected using the Escort IV reagent (Sigma) with 3µg of a Btd expression vector or with a pAc control expression vector. The medium was replaced the next morning, and cells were harvested 36–48 h post-transfection. The expression of Btd was verified by western blot (Fig. S5) with anti-V5 antibodies (Life Technologies). Cell extracts were prepared from 1.5×10^6 to 1.6×10^6 cells overexpressing Btd (using 50mM Tris pH 8.0, 150 mM NaCl, 5mM EDTA and 0.5% NP40) for loading onto the device.

2. Figures

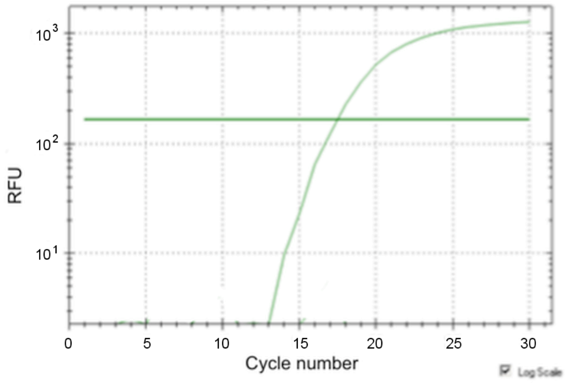

Figure S1: Calculation of qPCR optimal cycle numbers (OCN). qPCR cycles were performed for a sample of the oligomers eluted from each SELEX round. The fluorescence signal obtained from the fluorescently labelled primers was plotted on a log-scale graph against the cycle number until a plateau was reached. The optimal fluorescence (horizontal line) was, in most cases, observed after 15-20 PCR cycles, and a plateau was observed after 30-40 PCR cycles. The original sample was then amplified by the number of cycles determined according to an optimal fluorescence level, below the plateau.

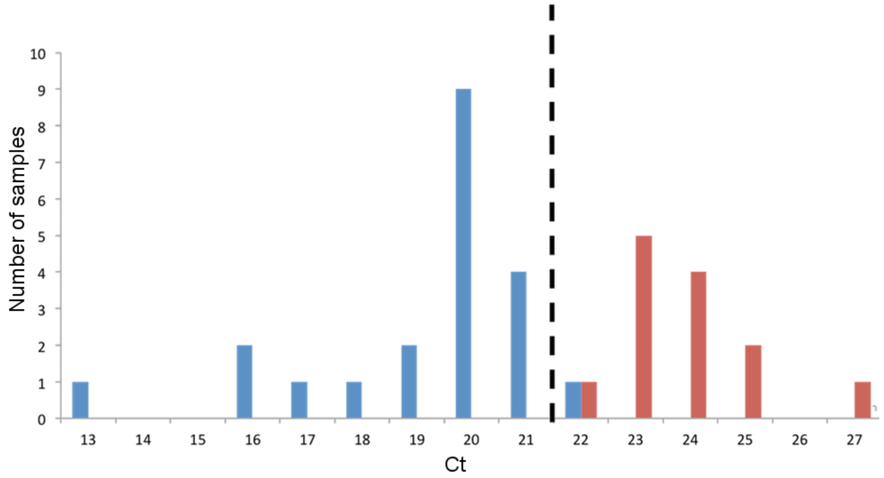

Figure S2: Comparison of qPCR for specifically and nonspecifically bound DNA. The histogram shows DNA amplification using qPCR of eluted DNA following TF binding (blue) compared to that of negative controls (no TF, red). The negative controls are used to measure non-specific DNA binding to the device comprising biotin, avidin and a TF-specific antibody. Following amplification, the Ct of each sample is compared. The OCN (black dashed line) is then determined as the cycle in which there is exponential amplification of specifically bound DNA, prior to amplification of non-specific oligomers.

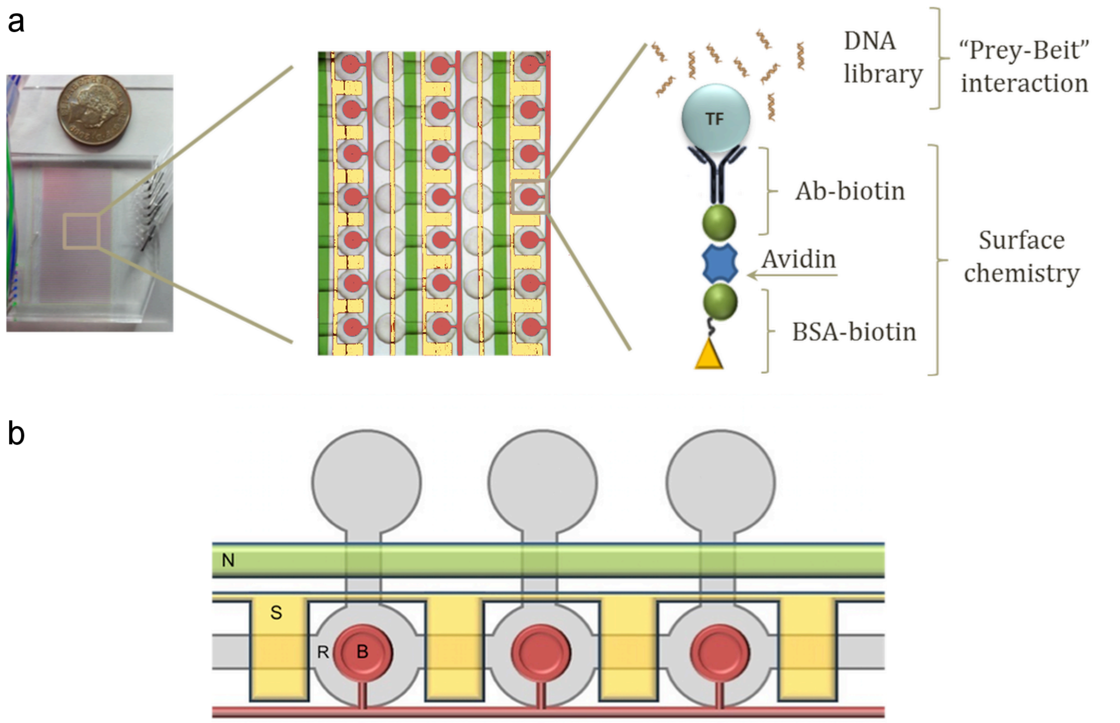

Figure S3: Microfluidic chip design and mechanics. (a) Chip and experimental design. The microfluidic device was fabricated with two layers of channels, the "control" layer (colored) and the "flow" layer (grey). The control layer controls the channels through which the experimental substances were flowed by applying mechanical pressure on the "flow" layer beneath. The "1K" chip comprises 16 individually addressed channels, each of which leads to 64 reaction chambers. This allows for 1024 individual reaction chambers per experiment, each comprising the desired chemical interactions (described in main text). **(b) Chambers and mechanical control.** The "neck" (N) isolates the reaction chamber (R) from an additional chamber (not required for the procedures described in the current manuscript). The "sandwich" valves (S) separate reaction chambers from each other, allowing for separate reactions to occur in each chamber. The "button" (B) applies pressure in the reaction chamber, which traps the substances present beneath it, and assists in washing of surface bound proteins in the reaction chamber. The average height of the chamber is 10 μm, and average chamber volume is less than 1 nl.

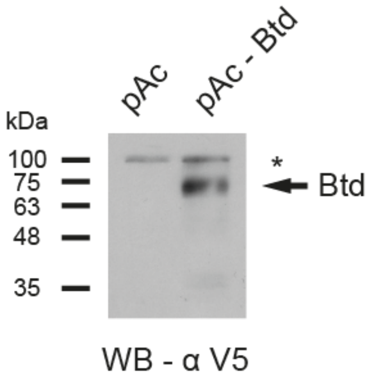

Figure S4: Western blot verification of Btd expression. *Drosophila* S2R+ cells transfected with a Btd-V5-His expression vector express a ~75 kDa protein. Protein extracts prepared from cells transfected with either a Btd expression vector or with a control pAc vector were subjected to western blot analysis with anti-V5 antibodies (Life Technologies). The asterisk denotes a cross-reactive band.
